# Point and counterpoint: Polatuzumab vedotin in the front-line therapy for diffuse large B- cell lymphoma

**DOI:** 10.3389/fonc.2022.1098375

**Published:** 2023-01-04

**Authors:** Colin Thomas, Sameep Thapa, Connor McLaughlin, Molly Halloran, Pierluigi Porcu

**Affiliations:** ^1^ Department of Medical Oncology, Sidney Kimmel Cancer Center at Thomas Jefferson University Hospital, Philadelphia, PA, United States; ^2^ Department of Internal Medicine, Thomas Jefferson University Hospital, Philadelphia, PA, United States

**Keywords:** diffuse large B cell lymphoma, polatuzumab vedotin, front-line, R-CHOP, treatment

## Introduction

Diffuse large B-cell lymphoma (DLBCL) is the most common type of lymphoma in the U.S. (1), with an age- adjusted incidence rate of 7.25 per 100,000 person-years, based on SEER data, accounting for 25%-30% of all non-Hodgkin lymphoma cases (2). The number of incident DLBCL cases in the US is projected to increase from 29,108 to 32,443 between 2020 and 2025, with a total rate of increase of 11% (3). This estimate is based on the increase in the number of individuals in older age groups, who have the highest incidence of DLBCL.

The chemotherapy regimen containing rituximab, cyclophosphamide, doxorubicin, vincristine, and prednisone (R-CHOP) has been the standard first-line therapy in DLBCL for nearly two decades, primarily based on two randomized studies. A Phase III clinical trial (LNH98-5) by the GELA (Groupe d’Etude des Lymphomes de l’Adulte), comparing 8 cycles of R-CHOP to 8 cycles of CHOP in 399 DLBCL patients age 60- 80 showed that R-CHOP increased the complete response (CR) rate from 60% to 76%, the event-free survival (EFS) from 49% to 68%, and the overall survival (OS) from 68% to 83%, compared to CHOP (4). Superiority of R-CHOP to CHOP was also observed in the MInT Trial (MabThera International Trial) that randomized 823 patients 18 to 60 year-old with favorable (IPI 0-1), stage II-IV DLBCL (or bulky stage I) to 6 cycles of R-CHOP versus 6 cycles of CHOP (5). Overall survival was 95% for R-CHOP vs 86% for CHOP.

Unfortunately, nearly 40% of all DLBCL patients treated with R-CHOP will relapse or have primary refractory disease (1, 6). Multiple clinical trials have explored strategies to improve upon the outcomes of R-CHOP such as replacing rituximab with the second-generation anti-CD20 antibody obinutuzumab (7), increasing dose intensity (8), adding etoposide (9), or including novel targeted agents such as lenalidomide (10), bortezomib (11), or ibrutinib to R-CHOP (12); however, no significant clinical improvements have been shown from these studies, leaving R-CHOP as the standard of care.

The POLARIX trial (NCT03274492), is a randomized, international, double-blinded phase III study that compared a novel regimen containing polatuzumab vedotin (pola), rituximab, cyclophosphamide, doxorubicin, and prednisone (pola-R-CHP) to R-CHOP as front-line therapy for patients with DLBCL (13). The pola-R-CHP regimen replaces the anti-microtubule drug vincristine (*Oncovin*) in R-CHOP with the novel agent polatuzumab vedotin. Pola is an antibody-drug conjugate (ADC) targeting the B-cell surface marker CD79b, which is ubiquitously expressed in mature B-cell lymphomas (14). It is comprised of the anti-CD79b monoclonal antibody SN8 linked to a monomethyl auristatin (MMAE) payload, which acts as a microtubule inhibitor (15). Pola has demonstrated efficacy in relapsed/refractory (R/R) DLBCL as monotherapy, with an overall response rate (ORR) up to 56% and complete response (CR) rate up to 15% (16). In combination with bendamustine and rituximab (BR), Pola-BR improved CR rate (40% versus 17.5%), progression-free-survival (PFS) (9.5 months versus 3.7 months) and median overall survival (OS) (12.4 months versus 4.7 months) as compared to BR (17). Additionally, the combination of Pola with rituximab in R/R DLCBL achieved an ORR of 54% and CR rate of 21% in a small but encouraging study that enrolled 39 patients (18).

## The data

Between November 2017 and June 2019, the POLARIX trial randomized 879 adult (18-80 years) patients with untreated DLBCL at more than 200 centers in North America, Europe, Australia, and Asia. Patients had intermediate- or high-risk international-prognostic-indices (IPI 2-5) and ECOG performance status 0-2. With 1:1 randomization, 440 patients were assigned to the pola-R-CHP investigational arm and 439 patients to the R-CHOP control arm. Both treatments were given for 6 cycles of either pola-R-CHP or R- CHOP, plus two cycles of rituximab alone. Patients with both GC-type and ABC-type DLBCL (Nanostring), as well as patients with double and triple hit (DH/TH) DLBCL were eligible. Patients with a history of low grade B-cell lymphoma or CNS involvement were excluded. In both arms patients were allowed to receive CNS prophylaxis, per investigator choice, but high dose methotrexate (HD-MTX) was not allowed. Radiation therapy to sites of bulky (≥7.5 cm) or extra-nodal disease was allowed. Radiation therapy had to be pre-planned by the treatment center, documented before randomization and initiated within 8 weeks after last study drug and started after end of treatment PET/CT. Use of G-CSF was required for primary prophylaxis on all patients. Infection prophylaxis was allowed, according to investigator preference. The primary endpoint was investigator- assessed PFS (defined as progression, relapse, or death). Secondary endpoints were investigator- assessed event free survival (EFS), overall survival (OS), and disease free survival (DFS). Response was scored according to the Lugano criteria. Patients were imaged by PET/CT after cycle 4 and at end of therapy. Follow up imaging consisted of CT or PET/CT every 6 months for 2 years, then every 12 months for 3 years, for a total duration of surveillance imaging of 5 years.

The median age in the pola-R-CHP and R-CHOP cohorts were 65 and 66 years, respectively; 68.6% of patients from both cohorts were from Western Europe, United States, Canada and Australia; 89.3% of patients in the pola-R-CHP cohort and 88.2% of patients in the R-CHOP cohort had Ann Arbor stage III/IV disease. Of all patients enrolled, approximately 30% were younger than 60, 50% had ≥2 extra-nodal sites, 44% had bulky disease, 62% had high risk IPI (IPI 3, 4, 5). Approximately a third of the patients had ABC-type DLBCL, 38-40% had double expressor (DE) phenotype, and 6-8% had DH/TH DLBCL (13).

Patients on both arms received 99% of the planned dose intensity: only 9% did not complete pola-R-CHP and 12% did not complete R-CHOP. Less than 5% of the patients received radiation therapy (2.5% in the pola-R-CHP arm and 4.1% in the R-CHOP arm). CNS prophylaxis was given in 16% of the patients in the pola-R-CHP arm, and 19% of the patients in the R-CHOP arm. After a median follow-up of 28.2 months (as of June 28, 2021), the POLARIX trial demonstrated a statistically significant improvement in PFS in the pola-R-CHP arm over the R-CHOP arm (13). The 2-year PFS rate in the pola-R-CHP arm was 76.7% (72.7-80.8, 95% confidence interval [CI]) versus 70.2% (65.8-74.6, 95% CI) in the R-CHOP arm. Overall- survival (OS) at 2-years was not statistically different between the two arms: 88.7% (85.7-91.6, 95% CI) in the pola-R-CHP arm versus 88.6% (85.6-91.6, 95% CI) in the R-CHOP arm. Although the trial was not designed to compare PFS in patient subgroups, the study included a subgroup analysis of PFS according to demographics and disease characteristics. Notable subgroups that did not benefit with pola-R-CHP included younger (¾60) patients, patients with the germinal-center (GC) B-cell–like subtype of DLBCL, patients who had bulky disease, and patients who had lower IPI scores (IPI 2 vs 3-5)

## The motion

Pola-R-CHP should replace R-CHOP as the standard front-line treatment for selected patients with DLBCL.

## Point: In favor of the motion

The pola-R-CHP regimen is an efficacious front-line therapy for DLBCL that represents an advancement compared to R-CHOP and should be used in its place, notably, in selected patients. First and foremost, although there was no improvement in OS at 2-years, the POLARIX trial demonstrated a statistically significant improvement in PFS with pola-R-CHP over R-CHOP at 2-years. An increase in PFS at 2-years may help in avoiding the toxicities and costs of intensive salvage regimens, such as autologous stem cell transplants or CAR-T therapy (19, 20). Based on a patient’s preference and their planned life events, choosing a therapy that puts them at greater odds of not having progression of disease within 2-years of treatment is not a trivial benefit: this could mean a better chance of being off treatment within a 2-year time frame for, say, a wedding, the birth of a grandchild, or planned vacation, thereby translating into meaningful improvements in quality of life.

Additionally, the lack of improvement in OS at 2-years should not dissuade clinicians from considering this treatment regimen. With regard to survival, DLBCL is a curable disease (21), thereby, a statistically significant improvement in PFS at 2-years, a time point after which disease progression is notably less common, is not insignificant (22, 23). Long-term follow-up data will be necessary to better assess a survival advantage.

The lack of improvement in OS may have something to do with the study population. The study showed that 11.8% (n=52) of patients receiving R-CHOP and 10.7% (n=47) of patients receiving pola-R-CHP had stage I-II disease. Patients with early stage DLBCL tend to have excellent outcomes with R-CHOP therapy; they often fair just as well with fewer cycles of R-CHOP despite receiving six cycles of therapy in this trial (24). As a result, including this group of patients in the POLARIX trial may have impacted the overall efficacy of pola-R-CHP, since a difference in efficacy between Pola-R-CHP and R-CHOP for this sub- population would be more difficult to detect given the already excellent outcomes with R-CHOP (24).

The subgroup analysis brings into focus specific populations of DLBCL patients who may have benefited more from the pola-R-CHP regimen. About 62% of patients enrolled in the trial had stage IV disease and had a 2-year PFS of 72.6% in the Pola-R-CHP arm vs. 66.1% in the R-CHOP arm with a hazard ratio (HR) of 0.8, however, the 95% CI ranged from 0.6-1.1, which falls out of range of statistical significance, but suggests that patients with advanced staged DLBCL benefit more from pola-R-CHP as compared to R- CHOP.

A PFS benefit for the pola-R-CHP arm was also seen in patients older than 60 years of age, with IPI score 3-5, and those with activated B-cell (ABC) subtype DLBCL. About 62% of patients enrolled in the trial had an IPI score of 3-5 with a 2-year PFS of 75.2% in the pola-R-CHP arm vs. 65.1% in the R-CHOP arm with a hazard-ratio (HR) of 0.7 (0.5-0.9 CI 95%). The POLARIX trial had 221 ABC DLBCL patients, of whom 102 received pola-R-CHP and 119 received R-CHOP; the 2-year PFS rates were 83.9% and 58.8%, respectively, with a HR of 0.4 (0.2-0.6, 95% CI). This difference suggests that the ABC subtype of DLBCL, a difficult-to-treat subtype with inferior outcomes relative to the GC subtype (25), has better outcomes with the pola-R-CHP regimen. Of note, however, the subgroup analysis was not the primary objective and the study was not powered to detect differences in efficacy among these subgroups. In the subgroup analysis, patients without bulky disease had an improvement in 2-year PFS with pola-R-CHP (2- year PFS rate of 82.7% versus 70.7%, HR 0.6, 95% CI 0.4-0.8), but patients with bulky disease did not (2- year PFS rate of 69% versus 69.7%, HR-1.0, 95% CI 0.7-1.5). With that said, slightly fewer patients in the pola-R-CHP arm received pre-planned radiotherapy (2.5%) as compared to the R-CHOP arm (4.1%). It should also be mentioned that there were slightly fewer patients in the pola-R-CHP arm who received CNS prophylaxis (16.4%) as compared to the R-CHOP arm (19.6%), but whether this difference had an impact on the PFS of pola-R-CHP relative to R-CHOP is not known.

The improvement in PFS with pola-R-CHP compared to R-CHOP was not associated with significant additional toxicities. Overall, the safety profile of pola-R-CHP and R-CHOP were similar with 34% grade 3/4 adverse events with pola-R-CHP versus 30.6% with R-CHOP. While pola-R-CHP was associated with more febrile neutropenia compared to R-CHOP (13.8% versus 8.0%, respectively), the number of patients who had grade 3/4 infections, or had to discontinue one of the drugs due to infection or neutropenia, were similar between the two treatment arms. Additionally, dose adherence was also similar between the two groups with 6.2% of the pola-R-CHP arm versus 6.6% of the R-CHOP arm having to discontinue at least one drug due to adverse events. Interestingly, 4.4% in the pola-R-CHP group discontinued polatuzumab vedotin, whereas 5.0% in the R-CHOP group discontinued vincristine due to adverse events, primarily neurologic events.

## Counterpoint: Against the motion

The argument against replacing R-CHOP with pola-R-CHP in the frontline setting can be discussed in terms of both economic impacts and clinical impacts. The pola-R-CHP regimen will carry a higher cost compared to R-CHOP due to replacing vincristine with the more expensive polatuzumab vedotin (26); however, for the sake of this counterpoint, our focus will be primarily on the clinical impacts of pola-R- CHP so as to focus on the clinical evidence at hand and not the economic consequences of a shift in first- line therapy. Although the POLARIX trial achieved its primary objective, the improvement in PFS with pola-R-CHP must be interpreted in the context of absolute risk reduction: notably, the number needed to treat (NNT). In order to prevent 1 patient with DLBCL from having an event (progression, death, or other [subsequent therapy or biopsy proven residual disease]) within a 2-year time frame, 17 patients will need to have been treated with the pola-R-CHP regimen as compared to R-CHOP. The NNT with pola-R-CHP over R-CHOP for preventing 1 case of progression/relapse at 2-years is 21. With that said, the question needs to be asked if the differences in toxicities between the two treatment regimens justify treating 21 patients with pola-R-CHP to prevent 1 patient from having progressive disease at 2- years.

Although adverse events were generally similar between the pola-R-CHP and R-CHOP treatment groups, there were some noteworthy differences in toxicity favoring the R-CHOP regimen. In the pola-R-CHP arm, 17 (3.9%) of the patients had grade 3/4 diarrhea compared to 8 (1.8%) in the R-CHOP arm; 52 (12%) of patients receiving the pola-R-CHP regimen had grade 3/4 anemia compared to 37 (8.4%) receiving R-CHOP; and 60 (13.8%) of the patients in the pola-R-CHP group had febrile neutropenia as compared to 35 (8%) in the R-CHOP group. Not only is febrile neutropenia associated with increased mortality, but hospitalizations for febrile neutropenia in oncology patients are expensive with costs ranging from $13,000-$24,000 (27–29), which is another point to consider in terms of potential economic impacts of pola-R-CHP independent of polatuzumab vedotin simply being more expensive than vincristine.

Subgroup analysis in the intent to treat (ITT) population showed a PFS benefit in ABC subtype DLBCL, patients with an IPI score of 3-5, and patients old than 60 years of age. Given the exploratory nature of the analysis, the trial was not statistically powered to detect differences in efficacy among the different sub-groups, therefore, definitive conclusions from these observations cannot be draw. Importantly, the trial assessed cell-of-origin (COO) subtype *via* Nanostring Lymph2Cx. This gene expression assay is a parsimonious digital gene expression (NanoString)-based test assessing 20 different genes for COO assignment in formalin-fixed paraffin-embedded tissue (FFPET) (30). Unfortunately, the vast majority of clinical oncologists do not have routine access to the Lymph2Cx assay and instead utilize the far more common method of determining COO *via* immunohistochemical (IHC) staining. The Tally method and Hans algorithm are the two methods typically used in the clinic to determine COO *via* IHC staining.

When compared to the gold standard of gene expression assays, the Tally method and Hans algorithm have inadequate sensitivities and specificities (31–33). The inferiority of IHC use in COO determination compared to gene expression assays puts into question the real-world translatability of the PFS improvement found in the ABC DLBCL subtype analysis.

Finally, the fact that the POLARIX trial included patients with MYC and BCL2/BCL6 translocations (double-hit/triple-hit disease) instead of offering a glimpse of a potentially better alternative to R-CHOP, leaves us with the uncomfortable feeling that pola-R-CHP may in fact be worse in this subset. In the pola-R-CHP arm, 7.9% of patients (n=26) had double-hit/triple-hit disease compared to 5.7% (n=19) in the R-CHOP arm. Although this was a very small data set (N=45), the sub-group analysis showed a worse 2-year PFS in the pola-R-CHP arm as compared to R-CHOP for double-hit/triple-hit disease. A larger sample size would be required to draw any conclusions; however, it is suggests that, for the time being, pola-R-CHP should not be used for double-hit/triple-hit disease, which comprises 5-10% of all DLBCL cases (34).

Overall, assessing survival data at a longer follow-up, such as 4 or 5 years, will be important to determine if the improvement in PFS with pola-R-CHP eventually translates to OS benefit. The heterogeneity in efficacy among the subgroups, especially given that polatuzumab vedotin targets a surface marker ubiquitously expressed on mature B-cell lymphomas, underscores that more information is needed to determine which patients will most benefit from pola-R-CHP and if any subgroups (double and triple hit, for example) may actually fare worse. Finally, the decision to move polatuzumab vedotin to the front-line setting must come with the acknowledgment that this could potentially impact its use in subsequent lines of therapy, such as part of the salvage regimen where it is used in combination with bendamustine and rituximab (17).

## Conclusions

For patients who are 60 years of age or older, have unfavorable disease (IPI score of 3 or greater), or have ABC type DLBCL based on Lymph2Cx COO gene expression assay, there should be a discussion of using the pola-R-CHP regimen for front-line therapy. When choosing a front-line therapy for these patients, shared decision making with the patient and their family will be of high value. The benefit of improved PFS at 2-years will need to be weighed against the slight increase in certain toxicities with the pola-R-CHP regimen: namely, febrile neutropenia, anemia, and diarrhea. However, the lack of OS improvement at 2-years will also need to be stressed emphasizing that long-term survival data is not currently available. Lastly, we recommend that when determining patients who may potentially benefit from the pola-R-CHP regimen, IHC staining COO studies should be used with the caveat of their inaccuracy in determining COO relative to gene expression assays, as used in the POLARIX trial. As more clinical studies collect predictive data utilizing gene expression COO assays as opposed to IHC staining methods, practicing clinicians will increasingly be required to adapt to using gene expression assays in order to provide the best results for their patients. For patients with double and triple hit DLBCL, pola-R- CHOP does not seem to be a promising alternative, and for the time being these patients will continue to be treated with DA-EPOCH-R. Lastly, with regard to the new genetic subsets of DLBCL including MCD, BN2, N1 and EZB, the impact of this new regimen remains to be determined (35, 36).

## Author contributions

All authors listed have made a substantial, direct, and intellectual contribution to the work and approved it for publication.
